# Efficacy of Ketogenic Diets on Type 2 Diabetes: a Systematic Review

**DOI:** 10.1007/s11892-021-01399-z

**Published:** 2021-08-27

**Authors:** Delphine Tinguely, Justine Gross, Christophe Kosinski

**Affiliations:** 1 Service of Anesthesiology, Lausanne University Hospital (CHUV), Rue du Bugnon 46, Lausanne, Switzerland; 2 Service of Endocrinology, Diabetes and Metabolism, Department of Medicine, Lausanne University Hospital (CHUV) and University of Lausanne, Avenue de la Sallaz 8, 1011 Lausanne, Switzerland

**Keywords:** Type 2 diabetes, Glucose intolerance, Ketogenic diet, Keto, Very-low-carb diet, Atkins diet

## Abstract

**Purpose of Review:**

To assess the pleiotropic effects of ketogenic diets (KD) on glucose control, changes in medication, and weight loss in individuals with type 2 diabetes, and to evaluate its practical feasibility

**Recent Findings:**

KD results in improved HbA1c already after 3 weeks, and the effect seems to persist for at least 1 year. This is associated with a reduction in glucose-lowering medications. The weight loss observed after a short time period seems to be maintained with a long-term diet. Adequate support (supportive psychological counseling, enhancing positive affectivity, reinforcing mindful eating) is necessary to achieve a benefit and to assure adherence.

**Summary:**

Despite the documented decrease in HbA1, a definitive causal effect of KD remains to be proven. KD should be performed under strict medical supervision. Future research should clarify how compliance can be maximized and how ketosis can be optimally monitored.

**Supplementary Information:**

The online version contains supplementary material available at 10.1007/s11892-021-01399-z.

## Introduction

**Fig. 1:**
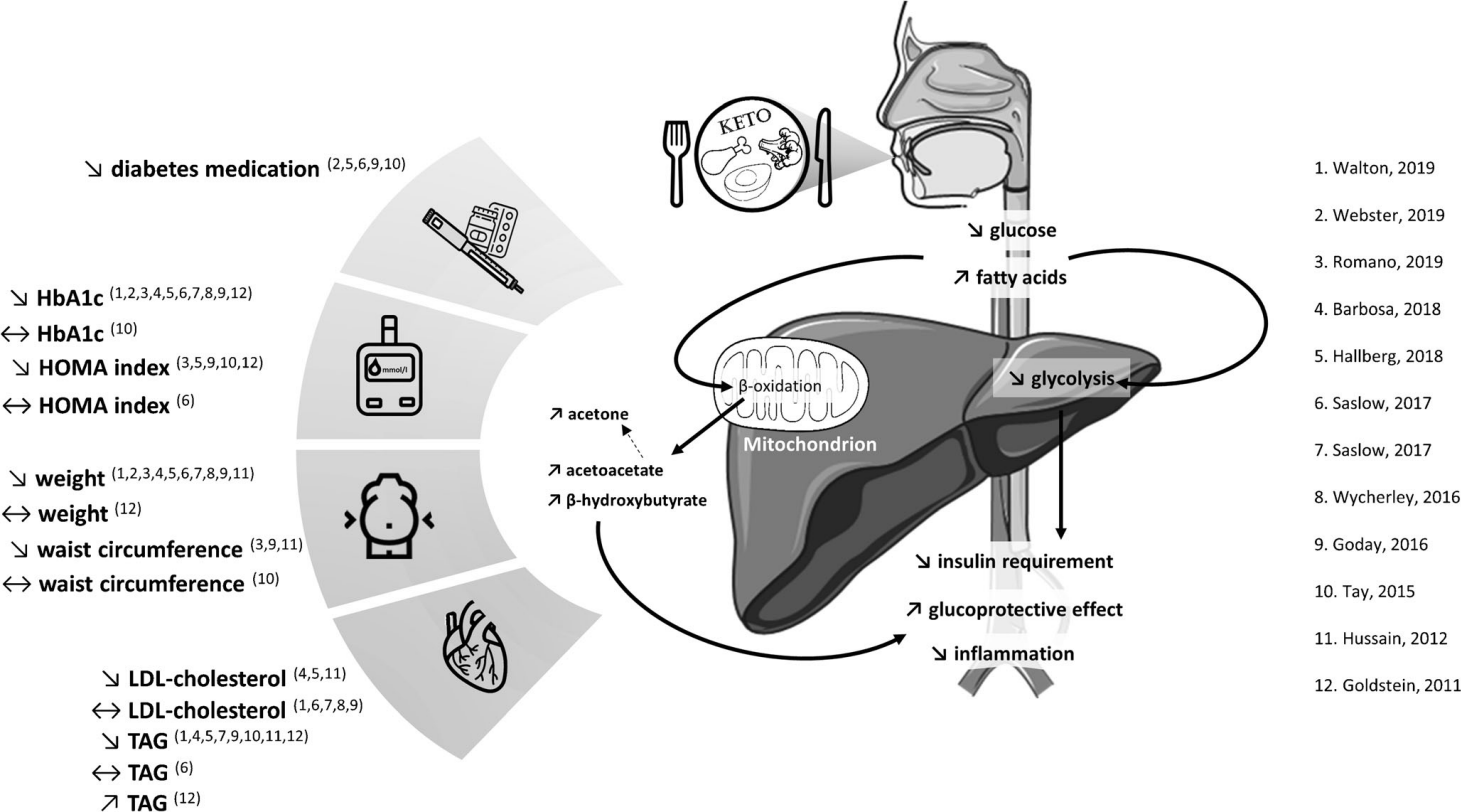
Potential pathophysiological mechanisms and metabolic effects of ketogenic diet in subjects with type 2 diabetes indicated by clinical trials [19–21 and 22–31]. Changes are indicated as: ↘, significant decrease; ↔, trend or no significant difference; ↗, significant increase. Abbreviations: HbA1c, glycated hemoglobin; FPG, fasting plasma glucose; HOMA, Homeostasis Model Assessment; LDL, low-density lipoprotein; TAG, triacylglycerol

The management of type 2 diabetes mellitus (T2D) includes lifestyle modifications that are combined with pharmacologic interventions as recommended by guidelines of international diabetes societies [1•, 2, 3]. Nutrition therapy guidelines often emphasize a reduction in the excessive amounts of carbohydrates, as well as limiting fat intake to be 20–35% of total calorie intake, with a focus on a decrease in saturated fats [4••]. Certain diets propose to reduce the carbohydrate intake even more drastically, in combination with a higher intake of fats, which become the most important source of calories. These regimens are referred to as ketogenic diets (KD) as they result in ketosis secondary to the severe carbohydrate restriction (<50 g/day) and the excess of free fatty acids. This combination induces a radical change in energy metabolism with an increase in fatty acid oxidation in the liver and production of ketone bodies [5]. These are acetoacetate (AcAc) and 3-β-hydroxybutyrate (BHOB), which are used as an energy source, and acetone, which is the product of spontaneous decarboxylation of AcAc [5]. Figure 1 depicts the pathophysiological mechanisms of the KD. In the last decades, KD have become increasingly popular, the most widely known is the Atkins diet [6], and some very-low-carbohydrate KD are even more restrictive with a carbohydrate intake <30 g/day [7, 8]. In subjects with type 2 diabetes, KD may be associated with positive effects on some cardiovascular risk factors [9–13]. Of note, the utilization of ketone bodies has shown protective cardiovascular effects in non-diabetic individuals [14], findings that need to be confirmed in individuals with diabetes mellitus. Despite these potentially positive effects, concerns have been raised about long-term adverse effects, particularly lipid metabolism and fatty liver disease, because of the high fat intake; yet recent studies did not corroborate these concerns [15, 16].

In this review, we discuss the pleiotropic effect of KD on glucose control including glycemic variability in individuals with T2D, its impact on the need for medications and body weight, and the practical feasibility.

## Methods

A systematic literature search was conducted in PubMed, Embase, and the Cochrane Central database covering 2011 to 2021 according to the PRISMA guidelines [17]. Search terms included the keywords “*Diabetes Mellitus*” OR “*diabetes*” AND “*Diet, Ketogenic*” OR “*ketogenic diet*” OR “*keto diet*” OR “*ketogenous diet*” OR “*ketotic diet*” OR “*very low carb diet*” OR “*very low carbohydrate diet*”. The search strategies for the databases are summarized in Supplementary Table 1. All publications in English published during the abovementioned period were included in the search. Trials with animals were not included. Studies were excluded if performed with children or adolescents, individuals with type 1 diabetes, and pregnant women, or if the diet was not ketogenic. Guidelines, case reports, reviews, meta-analyses, and abstracts were also excluded. The three authors independently screened the abstracts to check inclusion criteria. The complete text of all publications fulfilling the inclusion criteria was obtained and included in the review. Authors of the publications were not contacted.

Data were collected independently and then compared. In cases of discrepancies, the publication was discussed until agreement was reached. Records identified from citations in the selected studies were included in the review if they were fulfilling all inclusion criteria. The quality of clinical trials was evaluated based on the Cochrane Risk of Bias tool. Details about the systematic review process can be found in Supplementary Appendix.

## Results

After screening of 585 publications, a total of 14 studies were included (Supplementary Figure 1) [18–31]. 12 studies were clinical trials (8 randomized, 4 non randomized) and 2 were retrospective or observational studies. Table 1 summarizes the studies that assessed the effect of KD in subjects with T2D.

**Table 1:** Overview of the clinical trials (*n*=14)

**Observational studies**					
**Author, year**	**Aim**	**General characteristics at baseline**	**Diabetes characteristics at baseline**	**Study design**	**Major findings**
Wong et al, 2020 Canada [18]	To explore the experience of individuals living with diabetes who have followed or follow the KD	*n*=14 8 ♀/6 ♂ Caucasian BMI 31.5±5.1 kg/m^2^	T2D (*n*=11)/T1D (*n*=3)	Retrospective	KD has been followed for 6 to 19 months Main motivation to start was improve blood glucose control or to reduce/stop taking diabetes medications, weight loss, and diabetes reversal Benefits such as improved glycemic control, weight loss, and satiety have been reported by participants The main challenges were the lack of support from health-care providers and information sources Most participants could continue the KD for the rest of their lives
Webster et al., 2019 South Africa [19]	To describe the foods and characteristics of a LCHF “lifestyle” that was sustainable and effective for certain T2D patients in a real-world setting	n=28 14 ♀/14 ♂ BMI 30±6 kg/m^2^	T2D Median diabetes duration 7.4 years HbA1c 7.5 (6.5–9.5) %	Prospective duration 15±2 months Adult who currently followed an LCHF diet for at least 6 months	A majority of participants perceived reduced hunger and cravings LCHF diets are socially difficult to follow ↘ HbA1c (*p*<0.01), ↘ diabetes medications ↘ weight (*p*<0.001) Lack of support by their doctors which may involve suboptimal medical supervision
**Interventional studies**					
**Author, year**	**General characteristics at baseline**	**Diabetes characteristics at baseline**	**Diet(s)**	**Duration of the intervention**	**Major findings**
Walton et al., 2019 USA [20]	n=11 11 ♀ Caucasian BMI 36.3±1.4 kg/m^2^	T2D HbA1c 8.9±0.4% No medication	**VLCKD:** CHO ~5% (≤30g/day), ~20–25% of protein, ~70–75% of fat. Adherence was monitored with weekly plasma ketones tests	3 months	↘HbA1c (*p*<0.0001) ↘Weight (*p*<0.0001) ↗ HDL-c (*p*<0.005), ↘TAG (*p*<0.005), ↘TAG: HDL-c ratio (*p*<0.005), ↔ LDL-c ↔ AST, ↔ ALT
Romano et al., 2019 Italy [21]	n=20 10 ♀/10 ♂ BMI 37.1±6.8 kg/m^2^	T2D Diabetes duration 5.9±1.7 years HbA1c 7.3±1.1% Medication: none (*n*=8), metformin (*n*=15), SU (*n*=5), insulin (10)	**VLCKD** 5–10% of CHO (≤25g/day), 60–70% protein, 25–30% of fat	2 months	↘ HbA1c (*p*<0.0001), ↘ HOMA index (*p*<0.0001) ↘ Weight (*p*<0.001), ↘ BMI (*p*<0.001), ↘ WC (*p*<0.001), ↘ segmental (*p*<0.001), whole fat mass (*p*<0.001) ↘ AST (*p*<0.0001), ↘ ALT (*p*<0.0001)
Myette-Côté et al., 2018 Canada [22]	n=11 4 ♀/7 ♂ BMI 34.0±8.0 kg/m^2^	T2D Diabetes duration 6.4±4.3 years HbA1c 7.0±1.0% Medication: metformin (*n*=5), metformin + SU (*n*=2), metformin + GLP-1 (*n*=1), metformin + SU + DPP4 (*n*=1)	3 energy-matched diets **1. Low-fat low-glycemic index diet ~**55% CHO, 25% protein, 20% fat **2. VLCKD** <10% CHO, 25% protein, ~65% fat **3. VLCKD + exercise** <10% CHO, 25% protein, ~65% fat + 15 min of walking beginning 30 min after each meals	4 days	**Compared to baseline:** ↘ proinsulin for VLCKD (*p* = 0.001) and VLCKD + exercise (*p*=0.005), but not for low-fat low-glycemic index diet **Compared to low-fat low-glycemic index diet** ↘ mean glucose in the VLCKD with or without exercise (*p*≤0.001), ↔ time in hypoglycemia
Barbosa et al., 2018 Germany [23]	n=36 22 ♀/14 ♂ BMI 35.0±5.0 kg/m^2^	T2D HbA1c 8.9±0.4 %	**Hypocaloric VLCKD**: caloric reduction of 1200–1500 kcal/day. 5–10% CHO (≤40g/day), 20–30% protein, 60–70% fat **Hypocaloric low fat (LF)** 1000–1200 kcal/day, 50% CHO, 20% protein, <30% fat	3 weeks	**Compared to baseline, in the VLCKD group** ↘ HbA1c **in the VLCKD** (*p*<0.001) ↘ weight (*p*<0.001), ↘ total body fat (*p*=0.001), ↘ visceral adipose tissue (*p*=0.024), ↘ intra hepatic fat (*p*=0.003) **Compared to baseline, in the LF group** ↘ weight (*p*<0.001), ↘ total body fat (*p*<0.001), ↘ visceral adipose tissue (*p*<0.001), ↘ intra hepatic fat (*p*<0.001) **Compared to baseline, in both group** ↘ T-Chol (*p*≤0.001), ↘ LDL-c (*p*≤0.004), ↘ TAG in both group (*p*≤0.042)
Hallberg et al., 2018 USA [24]	*n*=349 (262 in intervention) 175 ♀/87 ♂ BMI 40.4±8.8 kg/m^2^ 18% African American	T2D Diabetes duration 8.44±7.22% HbA1c 7.6±1.5 Medication: metformin (*n*=15), SU (*n*=62), insulin (10)	Personalized CHO restriction, which provides 0.5–3.0 mmol/l of BHOB level in blood. Protein 1.5 g/kg of body weight Fat to satiety	12 months	↘ HbA1c (*p*<0.0001), ↘ FPG (*p*<0.0001), ↘ fasting insulin (*p*<0.0001), ↘ HOMA index (*p*<0.0001), ↘ diabetes medication, except metformin (↗) and GLP-1 (↔) ↘ weight (*p*<0.0001) ↘ TAG (*p*<0.0001), ↗ HDL-c (*p*<0.0001), ↘ LDL-c (*p*<0.0001), ↔ ApoB ↘ ALT (*p*<0.0001), AST (*p*<0.0001)
Saslow et al., 2017 USA [25]	n=34 BMI >25 kg/m^2^	T2D HbA1c >6.0% No insulin ≤3 glucose-lowering agents	**VLCKD:** non-calorie-restricted diet, CHO 20–50 g/day (0.5–3.0 mmol/l of plasma BHOB level) **Medium CHO, low fat:** 500 kcal reduced/day, CHO 45–50%, low fat	12 months	**Compare to control group, VLCKD** ↘ HbA1c (p<0.007), ↔ fasting insulin, ↔ HOMA index ↘ medication (SU and DDP-4 inhibitors, *p*=0.005, metformin *p*=0.08) ↘ weight (*p*<0.001) ↔ TAG, ↔ HDL-c, ↔ LDL-c
Saslow et al., 2017 USA [26]	n=25 15 ♀/10 ♂ Different types of ethnic (60% Caucasian)	T2D Mean diabetes duration ≈ 5 years Mean HbA1c ≈ 7.0%	**VLCKD:** CHO 20–50 g/day **“Create Your Plate” diet:** low-fat diet, lean protein sources (1/4 of plate), and limited starchy (1/4 of plate) and non-starchy vegetables (1/2 plate)	8 months	**Compared to the control group, VLCKD** ↘ HbA1c (*p*=0.002) ↘ weight (*p*<0.001) ↘ TAG (*p*=0.01), ↔ HDL-c, ↔ LDL-c
Wycherley et al., 2016 Australia [27]	n=115 49 ♀/66 ♂ BMI 34.6±0.4 kg/m^2^	T2D HbA1c 7–10%	**Both diet was energy reduced** **VLCKD (*n*= 58):** CHO 14%, 28% protein, 58% fat **High CHO Diet (*n*=57):** CHO 53%, 17% protein, 30% fat	12 months	**In both group; no different effect of diet** ↘ HbA1c (*p*<0.001) ↘ weight (*p*<0.001)
Goday et al., 2016 Spain [28]	n=89 58 ♀/31 ♂ BMI 33.1±1.6 kg/m^2^	T2D HbA1c 6.9±1.1% No insulin 80% glucose-lowering agents 20% lifestyle	**VLCKD (*n*=45), three stages:** 600–800 kcal/day CHO <50 g, protein 0.8–1.2g/kg of ideal weight, fat (10 g of olive oil/day) **Standard low-calorie diet (*n*=44)** 500–1000 kcal, CHO 45–60%, protein 10–20%, fat <30%	4 months	**Compared to baseline, in both group** ↘ HOMA index (*p*≤0.001) ↘ BMI (*p*<0.0001), ↘ WC (*p*≤0.048) ↔ T-Chol, ↔ LDL-c, ↔ HDL-c **Compared to baseline, in the VLCKD diet group:** ↘ FPG (*p*<0.0001), ↘ HbA1c (*p*<0.0001), ↘ medication (*p*=0.0267) ↘ weight (*p*<0.0001) ↘ TAG (*p*<0.004)
Tay et al., 2015 Australia [29]	*n*=115 BMI 34.6±4.3 kg/m^2^	T2D HbA1c 7.3±1.1 % Duration of diabetes 8±6 years	**VLCKD (*n*= 57):** hypocaloric. CHO 14% (50 g/d), protein 28%, fat 58% (10% saturated fat) **HC diet (*n*=58):** energy-matched, CHO 53%, protein 17%, fat 30% (10% saturated fat) + physical activity (60 min; 3 day/week)	12 months	**Compared to baseline, in both group** ↔ HbA1c, ↔ FPG ↔ BMI, ↔ WC **Compared to HC diet group, VLCKD** ↔ MAGE (*p*=0.09), ↘ CONGA-1 (*p*=0.003), ↘ CONGA-4 (*p*=0.02), ↔ time in euglycemia, ↔ time in hypoglycemia, ↘ medication (*p*=0.02) ↘ TAG (*p*=0.001), ↗ HDL-c (*p*=0.002)
Hussain et al., 2012 Kuwait [30]	*n*=363 277 ♀/86 ♂ BMI 37.3±0.3 kg/m^2^	Non-diabetic (261) and T2D (102) HbA1c 7.9±0.1% With the diet initiation, antidiabetic medications were decreased	2 groups attributed on personal preferences. **VLCKD (*n*=143),** initial goal CHO ~ 20 g/day **LCD (*n*=220),** 2200 kcal/day	6 months	**In the T2D subgroup and compared to baseline, in both group** ↘ weight (*p*<0.0001), ↘ WC (*p*<0.0001) **In the T2D subgroup and compared to LCD, VLCKD diet group** ↘ TAG (*p*<0.0001), ↘ T-Chol (*p*<0.0001), ↘ LDL-c (*p*<0.0001), ↗ HDL-c (*p*<0.0001)
Goldstein et al., 2011 Israel [31]	n=52 27 ♀/25 ♂ 35–75 years BMI 30–39.9 kg/m^2^	T2D HbA1c >7% Diet or oral medication	Stage 1 (4 weeks): Dietary Approaches to Stop Hypertension (DASH) diet with 20% kcal restriction Stage 2 (3 months): 1. **Atkins diet no caloric restriction (*n*=26):** <25g CHO/day for 6 weeks and after, ≤40g/day 2. **ADA calorie-restricted diet (*n*=26):** ♂ ≥1500 kcal/day, ♀ ≥1200 kcal/day. CHO 40–45%, protein 20%, 35g fibers	3–12 months	**Compared to the end of stage 1, in both group** ↘ HbA1c, ↔ FPG ↔ weight ↗ TAG (*p*=0.027), ↘ T-Chol (*p*=0.038), ↗ HDL-c (*p*=0.0026)

Data are presented as mean±SD, or mean (range), or as *n* (%). *p*-value is indicated for changes (↗ or ↘) in brackets if provided by the authors.

Abbreviations: *KD*, ketogenic diet; *T2D*, type 2 diabetes mellitus; *T1D*, type 1 diabetes mellitus; *HbA1c*, glycated hemoglobin; *FPG*, fasting plasma glucose; *HOMA*, Homeostasis Model Assessment; *SU*, sulfonylurea; *LCHF*, low CHO high fat; *VLCKD*, very low calorie ketogenic diet; *BMI*, body mass index (kg/m^2^); *WC*, waist circumference; *OW*, overweight subjects; *OBW*, obese weight subjects; *ADA*, American Diabetes Association; *CHO*, CHO; *AST*, aspartate transaminase; *ALT*, alanine transaminase; *T-Chol*, total cholesterol; *LDL-c*, low-density lipoprotein cholesterol; *HDL-c*, high-density lipoprotein cholesterol; *TAG*, triacylglycerol; *ApoB*, apolipoprotein B; *BHOB*, beta-hydroxybutyrate; *MAGE*, mean amplitude of glycemic excursion; *CONGA-1*, continuous overall net glycemic action of observations 1 h apart; *CONGA-4*, continuous overall net glycemic action of observations 4 h apart

### Effects on Glucose Control

Ten out 14 included studies showed a positive impact on glycated hemoglobin (HbA1c) (Table 1). In short-term studies, HbA1c improvements were variable, with reductions of 0.6% after 3 weeks [23] to 0.9% after 4 months [28], or even 1.3% after 32 weeks [26]; in the latter study, 55% of the patients in the intervention group had an HbA1c <6.5% versus 0% in the control group (*p*=0.02) [26]. HbA1c reduction from 8.9 to 5.6% (*p*<0.0001) was also reported in another study after 90 days [20]. The chance of lowering HbA1c <7% was two-fold higher with KD compared to a standard hypocaloric diet [28]. In a further study, in which glucose-lowering medications were reduced before the diet, a significant reduction of the HbA1c level from 7.8 to 6.3% with KD has been reported after 24 weeks [30]. The HbA1c-lowering effect could be maintained in long-term follow-ups, with a significant reduction from 7.6 to 6.3% after 1 year, albeit most changes appeared in the first 70 days [24], or from 7.5 to 5.9% after 15 months [19]. The same authors even reported remission in 10 of 24 participants with T2D (HbA1c <5.7% and no medication) after 15 months [19]. Saslow et al. compared a very low-carbohydrate ketogenic diet (VLCKD) with a moderate-carbohydrate, calorie-restricted, low-fat diet (MCRC) administered for 12 months and found that the VLCKD group had a greater reduction in HbA1c levels than the MCRC group (VLCKD 6.6 to 6.1%; MCRC 6.9 to 6.7%) [25]. Some studies reported a significant decrease in HbA1c, but without differences compared to the control group. Tay et al., who compared an isocaloric very-low-carbohydrate high-fat to a high-carbohydrate low-fat diet found a comparable HbA1c decrease of 1% in both arms after 52 weeks [29]. A study from Goldstein et al. compared the Atkins KD to a conventional hypocaloric diet and found a similar decrease in HbA1c levels in both groups at 6 weeks, 3 months, 6 months, and 1 year, with no statistical differences between the groups [31]. Finally, a similar reduction in HbA1c after 12 months has been reported in a study comparing a very-low carbohydrate diet (LowCHO) versus a traditional isocaloric higher carbohydrate low fat diet (HighCHO) (LowCHO 7.2 to 6.3%, HighCHO 7.4 to 6.3%) (*p*<0.001) [27].

The glycemic variability seems to improve with a KD. Tay et al. demonstrated an improvement in blood glucose stability in the low-carb group, whose subjects spent more time in the euglycemic range (*p*=0.07) and were less frequently in the hyperglycemic range [29]. However, the proportion of time spent in the hypoglycemic range was similar in both groups [29]. KD are also associated with significant improvements in fasting plasma glucose and mean glucose levels, both in short-time and long-time studies [21, 22, 25, 28–30].

### Effects on the Use of Glucose-Lowering Medication

A reduction in the use of glucose-lowering medications subsequent to KD has been observed by several studies. In a study by Saslow et al., 60% of participants could discontinue sulfonylureas and/or DPP-4 inhibitors, and 30% metformin after KD, but none of the subjects could do so in the control group [25]. Tay et al. reported a greater reduction in glucose-lowering agents following KD compared to the control group (*p*=0.02) [29]. At the 1-year follow-up, Hallberg et al. documented a significant reduction for all diabetes medications in participants of the KD group compared with the usual intervention [24]. Specifically, the overall prescriptions (not including metformin) dropped from 57 to 30%; insulin therapy was reduced/interrupted in 94% of users, sulfonylureas were discontinued in 100% of users, and metformin decreased slightly (from 71 to 65%, *p*=0.04) in the intervention group [24]. In a study exclusively including patients on metformin, there were no changes in the dose between the study groups [26]. The authors suggested that this may be explained by the safety of metformin, which does not prompt rapid modifications of its dose [26]. Finally, Webster et al. reported a decrease in glucose-lowering medications, including discontinuation of insulin in 8 of 11 participants, after 15 months [19]. In many studies, the patients were asked to interrupt or decrease all medications before KD, which hampers the interpretation of the results [21, 30]. As even a modest weight loss can have a beneficial effect on glycemic control, a reduced drug use has been observed in both arm (KD and control diet) in several studies [29, 31].

### Effects on Weight

Changes in weight seem to be dependent of the duration of interventional trials. In short-term studies, significant changes in weight loss and body fat were reported after 3 weeks, yet both with KD and the weight-lowering regiment used in the control group [21, 23]. Romano et al. noticed a predominant reduction of abdominal fat mass with a preserved lean mass with a rigorous low-calorie KD during an 8-week intervention (15.77% at the end) [21]. In studies comparing hypocaloric diets, participants achieved a significant weight loss with the VLCKD compared to the control arm after 4 months (BMI 33.3 to 27.9 kg/m^2^; standard low-calorie diet BMI 32.9 to 31.0 kg/m^2^) (*p*<0.001) [28], as well as after 6 months of diet (VLCKD BMI-12% ; LCD BMI-6%) (*p*<0.0001) [30]. In long-term studies, some authors did not report any statistical differences between the groups after 12 months of follow-up, when comparing KD versus calorie-restricted diet [31], or LowCHO versus isocaloric HighCHO [27], nor changes in fat mass and waist circumference after 12 months [29]. In a study by Goldstein et al., a greater weight loss at 6 months was reported with KD, compared to a standard calorie-restricted diet, only in the participants with good dietary adherence (3.7 kg, *p*=0.026), as documented by the presence of urinary ketones, but this benefit did not persist at 12 months [31]. A descriptive study by Webster et al. reported a weight loss of 16 kg (*p*<0.001) that persisted after 15 months of KD [19]. Finally, Saslow et al. concluded that participants on VLCKD lowered their BMI more than patients in a moderate-carbohydrate, calorie-restricted, low-fat diet group (respectively 8.35% and 3.8%) after 12-months of follow-up [25]. Similarly, in a separate study by the same group, in which patients were accompanied with online support, 90% of the participants on KD lost 5% of their body weight compared to only 29% in the control group (*p*=0.01) [26].

### Effects on Lipids, Kidney, and Liver function

The effects of KD on lipid profile are heterogenous, with improvement of LDL-cholesterol [23, 24, 30] and triglycerides [20, 23, 24, 26, 28–31] reported by some authors, while others reported no significant change in LDL-cholesterol [20, 25–28] and triglycerides [25], or even an increase in triglycerides [31].

No significant changes in renal parameters (urinary albumin-to-creatinine ratio, estimated glomerular filtration rate, creatinine, and blood urea) were reported after 4 months [28] or 12 months of KD [31]. A study noticed an improvement of the glomerular function in the initial 70 days of follow-up [24].

Liver function tests, specifically alanine aminotransferase (ALT) and aspartate aminotransferase (AST), did not differ after 4 months of follow-up [28]. Significant reduction of AST and ALT was reported after 8 weeks [21] and 70 days [24].

### Adherence and Feasibility

Due to their restrictive pattern, KD may be difficult to follow in the long-term, but the adherence seems to be improved by psychological support, enhancing positive affectivity and reinforcing mindful eating. In the study by Saslow et al., the mean retention was 85.3% after 12 months, but as soon as the support diminished, the dropout rate raised [25]. Some authors reported a dropout rate of only 8% with personal support versus 46% if participants were simply accompanied online (*p*=0.07) [26]. The coaching for developing behavioral adherence strategies, including positive affect regulation and mindful eating strategies, seems to be decisive for the success of the intervention. Factors that may negatively affect adherence may be related to frustration about unreached goal such as improved glycemic control and/or weight loss. Importantly, a more robust psychological support clearly appears to improve dietary adherence [26]. A minimal level of personal attention (online or in person meeting) seems to be necessary to achieve a benefit [20]. Goldstein et al. demonstrated a progressive decrease in the adherence rate to meet the carbohydrate restriction target as documented by absent biological ketosis during 12 months of observation [31]. The monotony of the diet and the need to abstain from fruits and some types of fresh vegetables may explain the lack of adherence, especially during a long period, particularly in the Mediterranean area, where consumption of fruit and vegetables is very common. Furthermore, the weight loss seems to be limited to the first 6 months. Goldstein et al. suggested that the long-term compliance and effectiveness of KD for obese diabetes patients in a Mediterranean environment is low [31]. Other authors highlighted that a support from providers and peers may be necessary to reach good adherence to KD and achieve sustained nutritional ketosis [24]. The fact that some KD interventions permit eating fat to satiety can be considered as a further potential advantage [24]. With appropriate support, most participants achieved and maintained nutritional ketosis up to 1 year, indicating durable efficacy. BHOB can be used as daily biofeedback to monitor and improve adherence [24].

Wong et al. aimed at identifying the main reasons people chose to follow KD: improving blood glucose control or reducing diabetes medication appeared to be the main motivations, followed by weight loss and diabetes reversal [18]. The lack of support from health-care providers and the absence of evidence-based information were the biggest challenges to maintain good adherence [18]. The advent of immediate results and additional health benefits (improvements in cognitive abilities, reduction in chronic pain levels, improvements in energy levels and quality of sleep) help in maintaining the motivation to follow KD [18]. Some participants noted that, compared to other diets tried in the past, adherence to KD was easier, tastier, and overall more enjoyable [18]. The reduced hunger helped participants to be less obsessed with the thought of food [18]. Another challenge is related to restaurant visits or gathering with friends and family, where it is difficult to follow KD. In terms of side effects, many participants found them to be less severe and enduring than expected [18]. Weighing in on their experiences, individuals on KD felt that the positive effects were outweighing the difficulties associated with adhering to the diet [18]. Webster et al. evaluated the experiences of individuals with T2D with KD in a real-world setting [19]. KD remained unchanged during the study. Participants noticed changes in eating behavior, with a reduction of cravings for sweets and snacks. Many participants lost weight without feeling hungry. The absence of a need for measuring quantities or counting calories was perceived as an advantage. Reduction in medications or avoiding the need for insulin therapy seemed extremely empowering and motivating. The main difficulty consisted in the challenges associated with socializing. Many patients experienced an increase in physical activity in connection with an improvement in energy levels.

For this reason, the primary mechanism(s) underlying the positive health impacts remain to be defined, and they may be multifactorial [19].

The study by Goday et al. did not report any serious adverse effect during 52 weeks of follow-up [28]. Mild adverse effects included asthenia, headaches, nausea, and vomiting. These were reported by 80% of the VLCK diet subjects as compared with 41% of the control population (low-calorie diet). (*p*<0.001). These adverse effects decreased with time in the VLCK diet group. At the end of the study, constipation and orthostatic hypotension were the most commonly reported adverse events in the VLCK group (*p*<0.005). Dietary adherence was similar between both study groups [28]. LCKD seems to be safe for a long period in obese participants [30].

### Clinical Considerations

In aggregate, KD seem to have positive effects in T2D patients and persist in long-term trials, as illustrated in Fig. 1. However, the main drivers leading to improved clinical outcomes need to be better defined. Despite the great decreases in HbA1 found in several studies, the study designs do not allow to definitely prove that KD has a causal effect. Of note, all groups experienced weight loss, which may explain the impact on HbA1c levels independent of the type of diet, and all interventional groups were subject to intensive diet counseling, including lifestyle recommendations. Thus, the effect of the dietary intervention is difficult to separate from the impact of other lifestyle changes. It is therefore important that future studies with strong control groups that avoid biases such as exercise, calorie restriction, and intensive supportive coaching further explore the benefits of KD.

Patients with T2D and/or obesity have a pro-inflammatory state [32–34], and KD may have beneficial effects on inflammation and positively modulate cardiovascular risk factors. For example, BHOB, one of the serum ketones detected in abundance on KD, promotes a reduction in inflammation by inhibition of the NLRP3 (NLR family pyrin domain containing 3) inflammasome in lipopolysaccharide (LPS)-stimulated human monocytes, leading to a reduced production of interleukin-1 beta (IL-1beta) and interleukin-18 (IL-18) [35–37]. This observation was confirmed with clinical data reporting a reduction in inflammatory markers with KD [38].

There are certain limitations in the included studies. The presence of ketosis (by measuring plasma or capillary BHOB) was not evaluated in most studies. However, this point is essential in order to assess the effectiveness of the diet. In long-term studies, positive results were described, but without significant differences compared to controls. This could be explained by the calorie restriction in all dietary interventions. It also seems important to determine whether an optimal target population exists for the KD; factors to consider include the onset of diabetes (recent or long-lasting) and the type of glucose-lowering medications. Moreover, the impact of reintroducing carbohydrates into the diet has not been explored. Due to variability in the nomenclature of the diets, we may have missed some studies in our systematic review. KD are very low-carbohydrates diets, usually ≤30 g/day and, hence, are sometimes referred to as “low-carb diet”. This is a known limitation that remains a challenge until a clear definition for KD has been accepted.

The adherence to KD requires a considerable personal investment. Indeed, the patient must adhere to strict rules to reach a ketogenic state which can be difficult depending on the dietary habits. Moreover, pursuing KD can be unaffordable for some individuals with low income. The long-term adherence can be exhausting, particularly during moments of social contacts. Following KD can, in some instances, reflect an underlying eating disorder, which is more prevalent among patients with T2D, and health care providers have to be aware of this possibility. Last but not least, KD is not recommended for pregnant or lactating women.

Known risks associated with KD include nephrolithiasis, worsening of dyslipidemia, and hypoglycemic episodes if the glucose-lowering therapy is not adapted. In all cases, diabetic patients on KD should be under strict medical supervision because of its ability in substantially lowering blood glucose levels. Some practical recommendations for adapting diabetes medication have been published [39•]. Insulin, sulfonylureas, and glinides should be progressively reduced by about 50%; biguanides, DPP-4 inhibitors, and GLP-1 agonists should be considered as optional; SGLT2 inhibitors are associated with a risk of ketoacidosis in some T2D patients with relative insulin deficiency and should therefore be avoided during a KD.

Future studies should assess the effect of performing KD intermittently, although this may not permit achieving a ketotic state if the intervention is too short. It is conceivable that inducing intermittent phases of ketosis is beneficial. If this is the case, the duration of the ketotic state and the intervals between these episodes need to be defined. Moreover, an excess in calories and/or carbohydrates between these periods of KD would need to be avoided. Another key axis of research should address which patients are most likely to benefit from KD, in particular at the cardiovascular level. Furthermore, the effect of KD on glycemic variability needs to be explored. This is of particular interest because it is accepted that glycemic variability is an independent cardiovascular risk factor [40–43]. Based on the nutritional composition of KD, it is plausible that the glycemic variability is less pronounced compared to a carbohydrate-rich diet. If confirmed, this may provide an additional argument for a potential cardiovascular benefit of KD.

### Limitations

The current systematic review included only publications covering the last 10 years. Many of the included studies have a limited methodology, sometimes without a control group, and a high or unclear risk of biases. However, these limitations are inherent to interventional diet studies, which cannot be single- or double-blinded. We also included retrospective observational studies that cannot be evaluated for quality given their design. Finally, we acknowledge that the short period of coverage and/or the sample size may form additional limitations.

## Conclusions

KD seems a promising dietary intervention for the improvement of the glycemic control in patients with T2D. However, the benefits believed to be induced by generating a ketogenic state need to be corroborated with well-planned research studies. KD should be accompanied with a structured support by dieticians and dedicated physicians to avoid adverse effects and to adjust glucose-lowering medications. Moreover, intensive support optimizes long-term adherence, which seems to be key to success.

## Supplementary information

ESM 1 (PDF 403 kb)
